# Phenylketonuria: reduced tyrosine brain influx relates to reduced cerebral protein synthesis

**DOI:** 10.1186/1750-1172-8-133

**Published:** 2013-09-04

**Authors:** Martijn J de Groot, Marieke Hoeksma, Dirk-Jan Reijngoud, Harold W de Valk, Anne MJ Paans, Pieter JJ Sauer, Francjan J van Spronsen

**Affiliations:** 1 Department of Metabolic Diseases, Beatrix Children’s Hospital, University Medical Center Groningen, University of Groningen, Groningen, the Netherlands; 2 Laboratory of Metabolic Diseases, Department of Laboratory Medicine, University Medical Center Groningen, University of Groningen, Groningen, the Netherlands; 3 Center for Liver, Digestive and Metabolic Diseases, University Medical Center Groningen, University of Groningen, Groningen, the Netherlands; 4 Department of Internal Medicine, University Medical Center Utrecht, Utrecht University, Utrecht, the Netherlands; 5 Department of Nuclear Medicine and Molecular Imaging, University Medical Center Groningen, University of Groningen, Groningen, the Netherlands

**Keywords:** Phenylketonuria, Phenylalanine, Tyrosine, Blood–brain barrier, Cerebral protein synthesis, Positron emission tomography

## Abstract

**Background:**

In phenylketonuria (PKU), elevated blood phenylalanine (Phe) concentrations are considered to impair transport of large neutral amino acids (LNAAs) from blood to brain. This impairment is believed to underlie cognitive deficits in PKU via different mechanisms, including reduced cerebral protein synthesis. In this study, we investigated the hypothesis that impaired LNAA influx relates to reduced cerebral protein synthesis.

**Methods:**

Using positron emission tomography, L-[1-^11^C]-tyrosine (^11^C-Tyr) brain influx and incorporation into cerebral protein were studied in 16 PKU patients (median age 24, range 16 – 47 years), most of whom were early and continuously treated. Data were analyzed by regression analyses, using either ^11^C-Tyr brain influx or ^11^C-Tyr cerebral protein incorporation as outcome variable. Predictor variables were baseline plasma Phe concentration, Phe tolerance, age, and ^11^C-Tyr brain efflux. For the modelling of cerebral protein incorporation, ^11^C-Tyr brain influx was added as a predictor variable.

**Results:**

^11^C-Tyr brain influx was inversely associated with plasma Phe concentrations (median 512, range 233 – 1362 μmol/L; delta adjusted R^2^=0.571, p=0.013). In addition, ^11^C-Tyr brain influx was positively associated with ^11^C-Tyr brain efflux (delta adjusted R^2^=0.098, p=0.041). Cerebral protein incorporation was positively associated with ^11^C-Tyr brain influx (adjusted R^2^=0.567, p<0.001). All additional associations between predictor and outcome variables were statistically nonsignificant.

**Conclusions:**

Our data favour the hypothesis that an elevated concentration of Phe in blood reduces cerebral protein synthesis by impairing LNAA transport from blood to brain. Considering the importance of cerebral protein synthesis for adequate brain development and functioning, our results support the notion that PKU treatment be continued in adulthood. Future studies investigating the effects of impaired LNAA transport on cerebral protein synthesis in more detail are indicated.

## Background

Phenylketonuria (PKU; OMIM 261600) is an inborn error of metabolism characterized by the inability to convert phenylalanine (Phe) to tyrosine (Tyr), caused by the deficiency of hepatic phenylalanine hydroxylase (EC 1.14.16.1). Untreated, PKU results in markedly elevated blood Phe concentrations, low-to-normal blood Tyr concentrations, and severe mental retardation [1]. The cornerstone of current treatment is reducing blood Phe concentrations, achieved in most patients by dietary Phe restriction combined with a Phe-free amino acid mixture [1]. When initiated early in life and followed continuously, this treatment prevents mental retardation [1], but mild reductions in intelligence quotient and impaired executive functioning remain [2-8]. Although it is clear that blood Phe concentrations throughout life closely relate to cognitive outcome in PKU [1], the pathophysiological consequences of elevated blood Phe concentrations are unknown [9-11].

Among others, reduced cerebral protein synthesis (CPS) is a particularly important pathophysiological mechanism [9-11]. CPS plays an essential role during cognitive development [12,13], and reduced CPS results in cognitive deficits in several disorders [13,14]. Our group was the first to study CPS in PKU patients [15]. In this exploratory study, ^11^C-Tyr cerebral protein incorporation was analyzed in five early and continuously treated PKU patients, showing a tendency for reduced CPS at increasing blood Phe concentrations. In a follow-up paper describing sixteen patients, we showed that CPS indeed decreases significantly with increasing blood Phe concentrations [16].

Reduced CPS in PKU is believed to result from impaired transport of large neutral amino acids (LNAAs) across the blood–brain barrier (BBB). These LNAAs (Phe, valine, isoleucine, leucine, Tyr, tryptophan, threonine, histidine, and methionine) compete for transport by the LNAA type I (LAT1) transporter [17-20], for which Phe has the highest binding affinity [17-19]. Possibly, competition between LNAAs for blood-to-brain uptake also occurs at other transporters, such as SLCA19 and SLCA15 [21]. An elevated blood Phe concentration reduces uptake of non-Phe LNAAs from blood to brain in several fundamental studies [17-20]. Moreover, similar findings have been obtained in clinical studies. Reduced uptake of ^75^Se-selenomethionine has been reported in nine late-treated mentally retarded PKU patients with blood Phe concentrations mostly >1000 μmol/L, compared to mentally retarded patients without PKU [22]. More recently, Landvogt et al. (2008) reported on the striatal decarboxylation of 6-[^18^F]-L-dihydroxyphenylalanine (FDOPA) in seven early and continuously treated PKU patients, with blood Phe concentrations between 510 and 1290 μmol/L. FDOPA and LNAAs share the same transporter. Since arterial FDOPA concentrations were not measured in this study, FDOPA transport from blood to brain could not be modelled directly. However, an indirect approach assessing FDOPA transport, based on striatal FDOPA signal intensity changes during the first six minutes of the study, suggested reduced FDOPA transport from blood to brain [23].

The question arises whether impaired LNAA transport across the BBB is associated with the decrease in CPS observed in PKU patients. Thus far, no studies have simultaneously assessed LNAA BBB transport and CPS in PKU patients. Accordingly, we investigate the hypothesis that the observed CPS reduction in PKU patients is associated with impaired LNAA BBB transport.

## Patients, materials and methods

### Study cohort

The study cohort is the same as that in Hoeksma et al. (2009) [16]. Sixteen adult PKU patients (seven males; age 16 – 47 years, median age 24 years) were included. Most patients (13/16) had been diagnosed by neonatal screening and were continuously treated since. Three patients were late diagnosed, one of whom was identified at 3 years of age, after his sister was diagnosed by neonatal screening. The other two late diagnosed patients were identified after diagnostic work-up for developmental delay at 6 and 9 years of age, respectively.

Aimed Phe treatment ranges were 200 – 500 μmol/L for all ages until 2002, according to Dutch national guidelines. From 2002 onward, aimed ranges were 120 – 360 μmol/L for patients <12 years and 120 – 600 μmol/L for patients ≥12 years [24]. Clinical severity of PKU was assessed by Phe tolerance, which is the amount of Phe that can be consumed daily while maintaining Phe concentrations within treatment range. Phe tolerance varied from 11 to 32 mg/kg/day at age 5 years, corresponding to 15% – 45% of the recommended daily protein intake for age-matched healthy subjects. Phe tolerance in early childhood reliably predicts Phe tolerance later in life [25].

At the time of study, all patients were non-pregnant without pregnancy wish, and free of concomitant diseases. The Medical Ethical Committee of the University Medical Center Groningen approved the study design. Informed consent was obtained in all participants.

### Isotope preparation

L-Tyr was obtained from Merck (Darmstadt, Germany). No-carrier-added L-[1-^11^C]-Tyr (^11^C-Tyr) was synthesized via microwave-induced Bucherer-Strecker synthesis, as described previously [26]. Next, ^11^C-Tyr was dissolved in a 0.9% (w/v) NaCl solution and passed through a sterile 0.22 μm Millipore filter. This process resulted in an average radiochemical yield of 800 MBq, a radiochemical purity >95%, and a specific activity >40 TBq/mmol. A dose of 400 MBq ^11^C-Tyr, corresponding to <10 nmol ^11^C-Tyr, was administered to each patient. The effects of this dose on Tyr metabolic fluxes are negligible [27].

### PET data acquisition

Data acquisition started after a 10 – 12 h overnight fast or a light breakfast with a very small amount of natural protein without amino acid supplement. Arterial and venous cannulae were inserted. A venous blood sample was taken to determine baseline plasma Phe and Tyr concentrations. Next, patients were positioned in the whole body PET scanner (ECAT EXACT HR^+^ PET camera, Siemens/CTI, Knoxville, TN, USA). Hereafter, 400 MBq of ^11^C-Tyr was infused in 8 ml 0.9% NaCl solution, followed by 32 ml solution to flush the injection line, using a programmable infusion system (Medrad International, Maastricht, the Netherlands). Total injection time was 50 s. During the next 50 min, a dynamic PET scan was performed. The scanner was used in two-dimensional mode, imaging 63 consecutive two-dimensional images of 128 x 128 pixels over an axial length of 15.5 cm. The in-plane resolution of the system was 4 – 5 mm full-width half-maximum, depending on the exact position in the plane. Spatial resolution was nearly isotropic in all three dimensions. The voxel size amounted to 16.5 mm^3^. The system was calibrated in absolute terms and cross-calibrated with an automated gamma well-counter (LKB Wallac, Turku, Finland). Throughout the scanning procedure, nineteen arterial blood samples were taken to measure plasma ^11^C-Tyr concentrations, as described by Willemsen et al. (1995) [27]: one sample every 10 s during the first minute after injection, one sample every 30 s during the next 4 minutes, and one sample at 10, 15, 20, 30, and 40 min. For each patient, plasma ^11^CO_2_ and ^11^C-protein concentrations were determined in nine samples, obtained at 0.5, 1, 2.5, 5, 10, 15, 20, 30, and 40 min after injection.

### Biochemical analyses

After baseline arterial blood collection, samples were centrifuged at 2000 g x 5 min at 4 °C, and plasma was transferred to a clean tube. Arterial plasma Phe concentrations were measured using the AccQ Tag® method (Waters BV, Breda, the Netherlands). Arterial plasma Tyr concentrations were measured by high-performance liquid chromatography with post-column ninhydrin derivatization on a Biochrome 20 amino acid analyzer (Pharmacia, Roosendaal, the Netherlands). Arterial plasma ^11^C radioactivity was determined with a calibrated and automated gamma well-counter. Quantification of ^11^C-Tyr, ^11^C-protein, and ^11^CO_2_ was performed as described by Willemsen et al. (1995) [27]. Plasma ^11^C measurements were corrected for radioactive decay (t_1/2_=20.4 min) during sample manipulation.

### Modelling of ^11^C-Tyr fluxes

**Figure 1:**
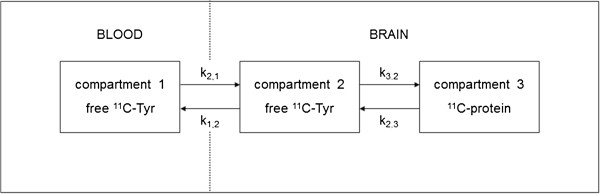
**Three-compartment model of **^**11**^**C fluxes, adapted from Willemsen *et al. *(1995).** Each rate constant k_A,B_ reflects the unidirectional ^11^C transport to compartment **A** from compartment **B**. Compartment 1: blood free ^11^C-Tyr; compartment 2: brain free ^11^C-Tyr; compartment 3: brain ^11^C-protein, i.e. ^11^C-Tyr incorporated into cerebral protein.

A three-compartment model was used to calculate ^11^C-Tyr fluxes (Figure 1), adapted from the five-compartment kinetic model described by Willemsen et al. (1995) [27]. This modification increases the accuracy of rate constant determination. The following three compartments were defined: (1) a blood compartment of free ^11^C-Tyr, (2) a brain compartment of free ^11^C-Tyr, and (3) a brain compartment of ^11^C-protein. With these three compartments, the following rate constants were defined: k_2,1_, describing the transport of free ^11^C-Tyr from blood to brain; k_3,2_, describing the incorporation of ^11^C-Tyr into ^11^C-protein in the brain; k_2,3_, describing the release of ^11^C-Tyr from ^11^C-protein in the brain; k_1,2_, describing the efflux of free ^11^C-Tyr from brain to blood.

This model was based on results obtained in animal studies, which indicate that certain metabolic ^11^C-Tyr pathways can be ignored within the time frame of our study [26,28]. ^11^C-Tyr can be metabolized to ^11^C-protein, ^11^C-3,4-dihydroxyphenylalanine (^11^C-DOPA), ^11^C-p-hydroxyphenylpyruvic acid (^11^C-HPPA), and ^11^C-p-hydroxyphenyllactic acid (^11^C-HPLA). ^11^C-HPLA can be metabolized to ^11^CO_2_ and next H^11^CO_3_^-^. Previous work on ^14^C-Tyr in rats has shown that 60 min after injection, the major metabolic pathway of ^14^C-Tyr is incorporation into ^14^C-protein [26]. In addition, the cerebral amount of ^14^C incorporated into non-protein metabolites, including ^14^C-DOPA, was <4% of the total cerebral amount of ^14^C [26]. Similarly, the amounts of brain ^14^CO_2_ and H^14^CO_3_^-^ were negligible [26]. In line with these results, Cumming et al. (1998) described that in rats, ^3^H-Tyr is mainly metabolized into ^3^H-protein rather than ^3^H-DOPA [28]. Another study investigating CPS by ^11^C-leucine incorporation [29] showed that within a time frame of 110 min, the contribution of ‘recycled’ ^11^C-leucine (i.e. leucine derived from proteolysis of ^11^C-protein reincorporated to ^11^C-protein) to the ^11^C-leucine incorporation rate is negligible.

Based on these studies, we made the following assumptions regarding the three-compartment model. First, ^11^C-Tyr is mainly metabolized to ^11^C-protein, and the contribution of brain non-protein ^11^C-metabolites (^11^C-DOPA, ^11^C-HPPA, ^11^C-HPLA, ^11^CO_2,_ and H^11^CO_3_^-^) to total brain radiation is negligible within the time frame of our study. Second, the contribution of ^11^C in the cerebral vasculature to brain ^11^C signal equals ~2.5% of total brain ^11^C radiation. Third, the effluxes of ^11^C-protein, ^11^CO_2_, and H^11^CO_3_^-^ from brain to blood, as well as k_2,3_, can be neglected within the time frame studied.

**Figure 2:**
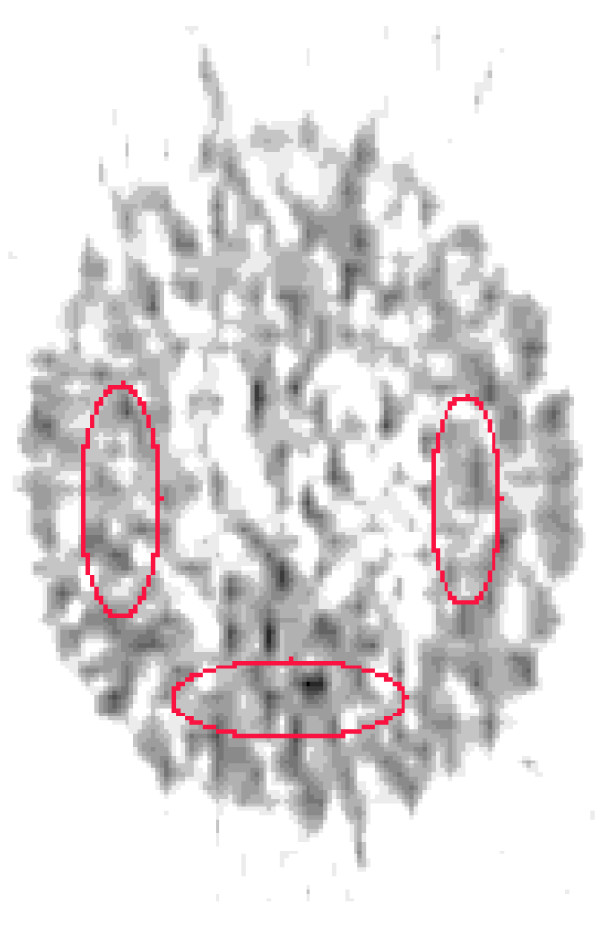
**Regions of interest for **^**11**^**C compartment and rate constant modelling.** Upper left: right temporal region, upper right: left temporal region, bottom: occipital region.

For the purposes of this study, k_2,1_ was considered to reflect transport of non-labelled LNAAs across the BBB, and k_3,2_ was considered to reflect CPS. Therefore, the primary parameters of interest in our study were k_2,1_ (the transport of ^11^C-Tyr from blood to brain) and k_3,2_ (the incorporation of brain ^11^C-Tyr into brain ^11^C-protein). Of note, ^11^C-Tyr incorporation into ^11^C-protein is not identical to net CPS, nor does it necessarily reflect incorporation of non-labelled, non-Tyr amino acids [27]. Both outcome parameters were assessed in three regions of interest (ROIs), as described by Hawkins et al. (1989) [29] and shown in Figure 2. This regional approach was chosen because compartment analysis cannot be performed on a pixel-by-pixel basis, due to the stochastic variation in radioactivity data [27]. Thus, the compartment model was fitted to the ^11^C radioactivity signal per ROI per sampling time point (C_tis_(t)), and to the arterial ^11^C-Tyr plasma radioactivity signal at each time point (C_pl_(t)), using the MATLAB software package (MATLAB version 5, MathWorks Inc., Natick, MA, USA).

### Statistical analyses

Testing for normality and homogeneity of variances was done using the Shapiro-Wilk test and Levene’s test, respectively. Normally distributed variables with homogeneous variances are reported as mean ± SD. Non-normally distributed variables and/or variables with inhomogeneous variances are given as range with median in parentheses.

Prior to multivariate analyses, multicollinearity was assessed by correlation analyses of predictor variables. Correlation coefficients |r| >0.80 were considered to reflect statistically relevant multicollinearity. For normally distributed variables with homogeneous variances, Pearson’s correlation test was used. Otherwise, correlation testing was done by Spearman’s rank correlation test. Multiple linear regression analyses were done using a stepwise approach, with either k_2,1_ or k_3,2_ as outcome variables. For both outcome variables, baseline plasma Phe, Phe tolerance, age, and k_1,2_ were used as predictor variables. For the k_3,2_ multiple regression analysis, k_2,1_ was additionally selected as a predictor variable. Baseline non-labelled plasma Tyr concentration was excluded as a predictor variable in all models, as it was involved in calculating ^11^C-Tyr fluxes and would thus by definition show high r-values.

Following multiple regression analyses, the assumptions of homoscedasticity, independence of errors, normality of error distribution, and linearity were evaluated. In addition, the presence of multicollinearity was again assessed, by analyzing variance inflation factor (VIF) and tolerance values. Statistical analyses were done using SPSS (Windows version 20, SPSS, Chicago, IL, USA). A two-sided p-value <0.05 was considered to be statistically significant.

## Results

### Patient characteristics and ^11^C-Tyr fluxes

Individual patient data are presented in Table 1. Baseline venous plasma Phe concentrations ranged from 233 to 1362 μmol/L (512 μmol/L). Five of the sixteen subjects studied had Phe concentrations above aimed treatment upper limit (i.e. 600 μmol/L). Baseline venous plasma Tyr concentrations ranged from 31 to 87 μmol/L (40 μmol/L). Mean rate of clearance of free ^11^C-Tyr by transport from blood to brain (k_2,1_) was 0.033 ± 0.010 ml plasma/g brain tissue/min. Mean ^11^C-Tyr incorporation into cerebral protein (k_3,2_) was 0.53 ± 0.21 nmol/g brain tissue/min. Mean rate of clearance of free ^11^C-Tyr by transport from brain to blood (k_1,2_) was 0.062 ± 0.014 ml plasma/g brain tissue/min.

**Table 1:** Study cohort characteristics

**Patient**	**Age (yrs)**	**Phe tolerance (mg/kg/day)**	**Plasma Phe (μmol/L)**	**Plasma Tyr (μmol/L)**	**k**_**2,1**_^**a**^	**k**_**3,2**_^**b**^	**k**_**1,2**_^**c**^
1	16	28	233	66	0.055	1.06	0.073
2	23	11	358	37	0.037	0.48	0.064
3	23	12	361	32	0.046	0.71	0.077
4	24	16	373	35	0.041	0.46	0.070
5	26	31	375	47	0.031	0.50	0.052
6	25	27	405	49	0.031	0.47	0.069
7	23	20	411	87	0.037	0.70	0.072
8	21	18	477	31	0.034	0.42	0.061
9	22	32	546	42	0.033	0.54	0.057
10	25	13	565	76	0.028	0.78	0.047
11	20	19	586	38	0.039	0.55	0.085
12	24	17	632	61	0.023	0.50	0.055
13	21	15	805	35	0.027	0.36	0.066
14	27	23	825	49	0.021	0.32	0.060
15	47	14	1078	33	0.019	0.21	0.029
16	33	12	1362	37	0.021	0.34	0.049

Phe tolerance values were obtained at age 5 years. Patients 15 and 16 were treated from 6 and 9 years of age onward, respectively. In these patients, Phe tolerance was determined 6 months after treatment start. Phenylalanine and tyrosine plasma concentrations shown are baseline values. ^a 11^C-Tyr transport from blood to brain (ml plasma/g brain tissue/min). ^b^^11^C-Tyr cerebral protein incorporation (nmol/g brain tissue/min). ^c^^11^C-Tyr transport from brain to blood (ml plasma/g brain tissue/min).

### ^11^C-Tyr transport from blood to brain

**Figure 3:**
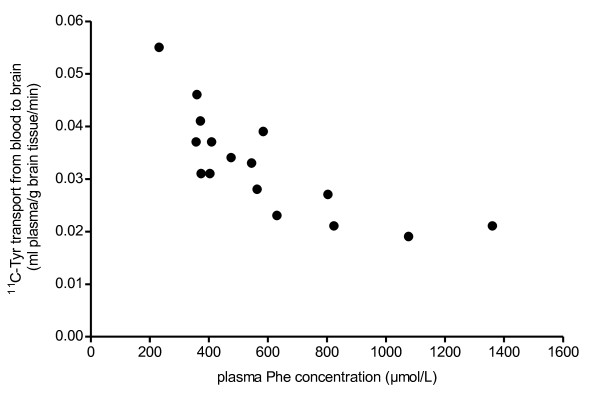
**Relation between **^**11**^**C-Tyr transport from blood to brain and plasma phenylalanine concentration.**

The stepwise multiple linear regression with ^11^C-Tyr transport from blood to brain as dependent variable resulted in a significant model (adjusted R^2^=0.669, F=16.182, p<0.001). Parameters of this model are summarized in Table 2. In this model, baseline Phe concentration and k_1,2_ were significant predictor variables for ^11^C-Tyr transport from blood to brain (Δ adjusted R^2^=0.571, p=0.013; Δ adjusted R^2^=0.098, p=0.041, respectively). ^11^C-Tyr transport from blood to brain showed a negative partial correlation with baseline Phe concentration, indicating that at increasing baseline Phe concentrations, ^11^C-Tyr influx to brain is decreased. ^11^C-Tyr influx to brain showed a positive partial correlation with k_1,2_, indicating that with increasing efflux of ^11^C-Tyr from brain to blood, ^11^C-Tyr transport from blood to brain increases. Statistical analyses to verify the assumptions for multiple linear regression showed that the assumptions of homoscedasticity, independent errors, normality of error distribution, and linearity had been met. Correlation coefficients between predictor variables, as well as VIF and tolerance values, were consistent with no multicollinearity. Figure 3 shows the relation between ^11^C-Tyr transport from blood to brain and plasma Phe concentration .

**Table 2:** **Multiple linear regression modelling for **^**11**^**C-Tyr transport from blood to brain**

	**B**	**SE B**	**β**	**Adjusted R**^**2**^**change**	**p**
Baseline Phe	−1.728 * 10^-5^	0.000	−0.529	0.571	0.013
k_1,2_	0.301	0.132	0.417	0.098	0.041
Constant	0.024	0.011	n.a.	n.a.	0.041

Multiple regression model with ^11^C-Tyr transport from blood to brain as outcome variable, using baseline plasma Phe, Phe tolerance, age, and k_1,2_ as predictor variables. Only statistically significant predictor variables are shown. k_1,2_: ^11^C-Tyr transport from brain to blood. SE: standard error.

### ^11^C-Tyr cerebral protein incorporation

**Figure 4:**
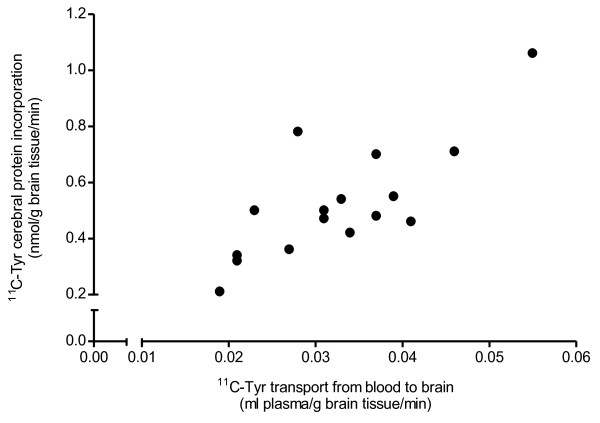
**Relation between **^**11**^**C-Tyr cerebral protein incorporation and **^**11**^**C-Tyr transport from blood to brain.**

The stepwise multiple linear regression with ^11^C-Tyr cerebral protein incorporation as dependent variable resulted in a significant model (adjusted R^2^=0.567, F=20.642, p<0.001), with only ^11^C-Tyr transport from blood to brain as a significant predictor variable. Parameters of this model are summarized in Table 3. ^11^C-Tyr cerebral protein incorporation showed a positive partial correlation with ^11^C-Tyr transport from blood to brain. Thus, lower ^11^C-Tyr influx values were associated with lower ^11^C-Tyr cerebral incorporation values. Following the main analysis, additional analyses were performed to assess the assumptions for multiple linear regression. These analyses showed that the assumptions of homoscedasticity, independent errors, normality of error distribution, and linearity had been met. Correlation coefficients between predictor variables, as well as VIF and tolerance values, were consistent with no multicollinearity. Figure 4 shows the relation between ^11^C-Tyr cerebral protein incorporation and ^11^C-Tyr transport from blood to brain.

**Table 3:** **Multiple linear regression modelling for **^**11**^**C-Tyr cerebral protein incorporation**

	**B**	**SE B**	**β**	**Adjusted R**^**2**^**change**	**p**
k_2,1_	16.303	3.588	0.772	0.567	<0.001
Constant	−0.008	0.122	n.a.	n.a.	0.949

Multiple regression model with ^11^C-Tyr cerebral protein incorporation as outcome variable, using baseline plasma Phe, Phe tolerance, age, k_1,2_ and k_2,1_ as predictor variables. Only statistically significant predictor variables are shown. k_2,1_: ^11^C-Tyr transport from blood to brain. SE: standard error.

## Discussion

This study is the first to simultaneously investigate LNAA transport from blood to brain and CPS in PKU patients. Our work can be summarized in two main findings. First, increased blood Phe concentrations are strongly associated with reduced ^11^C-Tyr transport from blood to brain. Second, reduced ^11^C-Tyr transport from blood to brain is strongly associated with reduced ^11^C-Tyr cerebral protein incorporation. Before discussing the results of this study, we will first address the strengths and limitations of our approach.

^11^C-Tyr transport from blood to brain and ^11^C-Tyr cerebral protein incorporation were calculated using a three-compartment model, adapted from [27]. This approach differs from that in our previous study [16], which only allowed for quantification of CPS. Our current model allows for the assessment of multiple compartments and ^11^C-Tyr fluxes between these compartments. As outlined above, this three-compartment model is based on results found in animal studies [26,28,29]. It should be noted that these findings have not been validated in humans.

The assumptions for performing multiple linear regression analyses were met, indicating that our conclusions can be generalized to other PKU patient cohorts. Still, for extensive statistical analyses such as regression modelling with four to five predictor variables, a cohort size of sixteen subjects is relatively small. This relatively small sample size may have reduced the power of the regression analyses in our study. Although the nonsignificant predictor variables in our study may correlate to some extent with their corresponding outcome variables, these correlations should be weaker that the currently observed correlations.

In order to acquire reliable measurements, a steady state condition in each patient was required. To obtain such a condition, patients fasted prior to the study and refrained from taking amino acid formula. After an overnight fast, some patients consumed a light breakfast with a very limited amount of Phe, whereas other patients remained fasted. We consider the influence of the light breakfast to be minimal, as we previously showed that such a breakfast does neither affect blood Phe nor blood Tyr concentrations [30]. Similarly, one could hypothesize that ceasing amino acid formula intake could have affected blood Phe and Tyr concentrations during the study period. First, intake of amino acid formula could reduce Phe uptake at the gut-blood barrier, thus reducing blood Phe concentrations. Second, formula intake could increase blood Tyr concentrations. Whether these effects would in turn influence ^11^C-Tyr fluxes remains to be investigated.

In the ^11^C-Tyr influx regression model, both baseline Phe and ^11^C-Tyr efflux from brain to blood were found to be significant predictor variables. Of these two variables, baseline Phe showed the strongest association with ^11^C-Tyr influx. This strong association supports the concept that increased blood Phe concentrations reduce binding of blood non-Phe large neutral amino acids to the LAT1 transporter, thus reducing brain influx of these amino acids. The observed relationship between increased blood Phe concentrations and reduced ^11^C-Tyr influx could not have been biased by *in vivo* tyrosine synthesis, as flux analyses were performed for ^11^C-Tyr rather than Tyr, and a standardized amount of ^11^C-Tyr was administered to each patient. The significant association between ^11^C-Tyr influx and ^11^C-Tyr efflux may be explained as follows. Similar to brain influx, brain efflux of large neutral amino acids is at least partly mediated by the LAT1 transporter [31]. In addition to LAT1 [32], other amino acid transport systems exist at the abluminal membrane of BBB endothelial cells, which similarly transport LNAAs in a competitive manner [33]. These transport systems may thus complement LAT1 in mediating LNAA efflux from brain to blood [31,33]. Therefore, increased brain Phe concentrations could competitively reduce ^11^C-Tyr efflux via several transporters, including LAT1. Such increased brain Phe concentrations likely occur at increased blood Phe concentrations, which in turn are significantly associated with reduced ^11^C-Tyr influx. Thus, reduced ^11^C-Tyr influx may be associated with reduced ^11^C-Tyr efflux. In the ^11^C-Tyr cerebral protein incorporation regression model, ^11^C-Tyr transport from blood to brain was found to be a significant predictor variable, whereas blood Phe concentration was not. Blood Phe concentration explained 57% of the observed variance in ^11^C-Tyr transport from blood to brain. Thus, 43% of the observed variance could not be prediced by blood Phe concentration. This relatively large variance unexplained by blood Phe concentration may clarify why blood Phe concentration did not independently predict ^11^C-Tyr cerebral protein incorporation.

Only two subjects had blood Phe concentrations outside the 360 – 1000 μmol/L range. On the one side, this result could limit the predictive value of our models for patients with blood Phe concentrations outside the studied range. Therefore, to further validate our conclusions, more patients with Phe concentrations <360 μmol/L and >1000 μmol/L at the time of study (presumably associated with relatively high and low values for LNAA BBB influx and CPS) should be investigated. On the other side, the blood Phe range studied offers new insights. First, contrary to previous reports, our dataset systematically studies LNAA BBB transport and CPS in PKU patients with blood Phe concentrations <1000 μmol/L. In the study of Oldendorf et al. [22], only two patients had blood Phe concentrations below this value. In the study of Landvogt et al. [23], individual blood Phe concentrations are not presented. However, it can be deduced from these data that at most three patients had blood concentrations <1000 μmol/L. Second, the blood Phe range in our study includes 600 μmol/L, the currently aimed treatment maximum for adults. Even in patients with blood Phe concentrations below this current treatment maximum, ^11^C-Tyr transport from blood to brain was decreased compared to that observed at lower blood Phe concentrations. This finding suggests that the current treatment maximum may have to be lowered in order to obtain optimal cognitive development.

The results presented in this study may have both fundamental and clinical implications. Regarding the fundamental relevance, reduced CPS may be one of the main mechanisms by which untreated PKU results in mental retardation. CPS is involved in many processes during brain development, including neuronal differentiation, maturation, migration, synaptic plasticity, and the formation of functional networks [12,13]. Thus, reduced CPS may negatively affect cognition via many mechanisms. Indeed, in inherited mental retardation diseases other than PKU, reduced CPS has been described [13,34]. Clinically, our data underline the importance of continuing PKU treatment after early adulthood. CPS-dependent developmental processes continue into the third decade [35,36], and CPS-dependent synaptic plasticity occurs lifelong [37]. Thus, also at adult age, reduced CPS could contribute to impaired cerebral functioning. Finally, the observation that both ^11^C-Tyr influx from blood to brain and cerebral protein incorporation are reduced in PKU patients considered to be well-treated, justifies the development of new treatments aiming to restore these abnormalities.

Follow-up studies should focus on the functional consequences of reduced CPS in PKU, as well as the identification of a safe blood Phe upper limit with regards to CPS. Another direction for future research is to identify the contributions of elevated brain Phe concentrations and reduced brain non-Phe LNAA concentrations to reduced CPS. Combination of PET with magnetic resonance spectroscopy allows for investigating the association between brain Phe concentrations and CPS.

In summary, we observed that reduced ^11^C-Tyr cerebral protein incorporation in early and continuously treated PKU patients is strongly associated with reduced ^11^C-Tyr transport from blood to brain. ^11^C-Tyr transport from blood to brain was significantly reduced at increasing blood Phe concentrations. These findings support the hypothesis that reduced CPS in PKU results from impaired LNAA transport from blood to brain. Our findings may serve as a starting point for both improving current treatment practice and developing new treatment strategies, in order to optimize cognitive outcome and quality of life in PKU patients.

## Abbreviations

LAT1: Large neutral amino acid type I; BBB: Blood–brain barrier; CPS: Cerebral protein synthesis; LNAA: Large neutral amino acid; PET: Positron emission tomography; Phe: Phenylalanine; PKU: Phenylketonuria; Tyr: Tyrosine.

## Competing interests

FJvS has received financial support from Nutricia and Merck Serono for research purposes and for advisory board membership. There are no additional potential sources of conflict of interest.

## Authors’ contributions

DJR, HWdV, AMJP, FJvS, and PJJS were involved in the study design and data collection. MJdG and MH performed the statistical analyses and wrote the manuscript, primarily supervised by FJvS. All authors were involved in critical appraisal of the results. DJR, AMJP, PJJS, and FJvS provided important contributions throughout the manuscript preparation process. All authors read and approved the final manuscript.
